# *Opuntia ficus-indica* as an Ingredient in New Functional Pasta: Consumer Preferences in Italy

**DOI:** 10.3390/foods10040803

**Published:** 2021-04-08

**Authors:** Nadia Palmieri, Alessandro Suardi, Walter Stefanoni, Luigi Pari

**Affiliations:** CREA Research Centre for Engineering and Agro-Food Processing, Via della Pascolare, 16, Monterotondo, 00015 Rome, Italy; alessandro.suardi@crea.gov.it (A.S.); walter.stefanoni@crea.gov.it (W.S.); luigi.pari@crea.gov.it (L.P.)

**Keywords:** consumer preferences, *Opuntia ficus-indica* (L.) Mill, functional food, pasta, Italy

## Abstract

*Opuntia ficus-indica* is a source of minerals and vitamins and has recently been used as ingredient to make a new functional variety of pasta. Italy was the first country in the world to produce pasta and is also the second largest producer of Opuntia in the world. According to an Italian sample, this study considers the main factors that could influence consumers when choosing functional pasta (featuring Opuntia) and characterizes distinct hypothetical consumer segments in terms of their food habits, pasta choices, and perceptions toward functional pasta featuring Opuntia. Data were collected using a web-based survey and with 328 respondents. Factor analysis (FA) with orthogonal rotation (varimax) was used to simplify the observed variables and hierarchical cluster analysis was performed with the FA results. Seven clusters were identified and the main results show that the level of education plays an important role in the perception of functional pasta. In fact, the perceptions of well-educated people differed from poorly-educated people. Moreover, the results showed significant respondent interest regarding health benefits and the nutritional and environmental aspects of functional pasta, which should encourage people’s acceptance and consumption of this new functional food. In addition, the respondent preferences reflect a value of experience towards the pasta, i.e., the belief of cooking typical Italian pasta. This means that Opuntia used for the production of functional pasta should maintain the organoleptic and physical properties of durum wheat-based pasta. In addition, respondent preferences for pasta featuring Opuntia could also be driven by its price.

## 1. Introduction

Functional foods have the appearance of traditional food but provides more benefits for human health and chronic disease prevention than conventional foods [1,2]. 

Several researchers [3,4,5] have considered the functional components of many conventional foods and have tried to develop new products [6,7]. Other authors have instead examined consumer acceptance and the perception of functional food products and have found [8] that socio-demographic and cognitive/attitudinal factors could hamper the demand for functional food. According to other authors [9,10,11,12,13], consumer acceptance for functional products is mainly determined by the perceived correlation between aspects such as diet and health [13]. In fact, consumers are conscious that some foods might prevent diseases and improve human wellbeing [14]. For these reasons there has been important growth in the market of functional food in Europe over the last decade [15,16]. In other words, functional food presents an interesting opportunity for firms [17,18] and the introduction of new products allows the exploration of new market opportunities in order to satisfy consumer needs and increase firm profitability in the food sector [13].

In this context, *Opuntia ficus-indica* is an important source of vitamins C, B1, B2, A, and E and minerals such as potassium, calcium, magnesium, and phosphorus [19], thereby presenting beneficial proprieties for human health with its consumption [13]. Moreover, it is used to treat diseases such as obesity, diabetes, arteriosclerosis, hypercholesterolemia, and cardiovascular disorders [20,21], and it also presents anti-inflammatory, antioxidant, hypoglycemic, antimicrobial, and neuroprotective properties [22].

At the global scale, Mexico represents the greatest cultivation of Opuntia, followed by Italy and then South Africa [23]. In Italy, Opuntia is mainly cultivated in Sicily and accounts for 7400 ha of cultivation at the national level and the production of 78,000 tons per year of fresh fruit, ranking Italy as both the second largest producer of Opuntia internationally [23] and the largest producer in Europe [24,25].

The fruit of Opuntia is eaten in both fresh food and processed forms due its strong organoleptic characteristics and high sugar content [19]. In fact, it is used to make food such as bread, nachos, tortillas, juice, jam [26], and biscuits [27]. Moreover, some authors [28] have tried to use Opuntia to make a new functional pasta and have shown that it could be considered a healthy food without altering the organoleptic and physical characteristics of the final food product [28]. Other authors [29] have instead studied the enrichment of durum wheat pasta with 3% Opuntia and have underlined that pasta with 3% Opuntia as an additive is a good functional food for maintaining a normal body weight and the prevention of age-related metabolic disorders [29]. 

As seen with the recent interest in the literature towards Opuntia as a food enrichment ingredient (e.g., [13,19,27,28]), with the health benefits of Opuntia [13,19,28], the common use of pasta in the Mediterranean diet [30,31], the daily pasta consumption for Italians [32], and Italian consumer preferences towards functional pasta, pasta featuring Opuntia may be an interesting prospect for investigation. 

To the best of our knowledge, no other study has been conducted concerning consumer preferences for pasta made from flour enriched with Opuntia. Thus, this study aims to fill this gap by analyzing consumer preferences for functional pasta with Opuntia in a sample of Italian consumers. In particular, the study aims to determine the main factors that could influence consumer choices regarding functional pasta and to characterize distinct hypothetical consumer segments in terms of their food habits, pasta choices, and perceptions toward functional pasta featuring Opuntia. In general, consumer preferences towards the attributes of pasta represents an important aspect for enterprises [30] to develop effective marketing strategies [32]. In fact, the profiles of consumer groups with similar needs are often used to develop marketing strategies [33]. In other words, the individualization of the preferences that drives a consumer in choosing functional pasta featuring Opuntia may represent an interesting chance for firms to identify new market niches in a country characterized by a typical Mediterranean diet [34,35].

## 2. Materials and Methods

### 2.1. Data Collection

The study data were collected using a web-based survey administered during the period of September–December in 2020. The survey was delivered through social media and e-mail as similarly performed in papers investigating consumer behaviors (e.g., [33,34,35,36,37,38]). Moreover, a snowball sampling recruitment method was also adopted in order to obtain a large number of participants [39]. The inclusion criteria were an age over 18 years, responsible for the grocery shopping in the family, and being willing to eat functional pasta containing Opuntia. From an initial sample of 350 consumers, 22 respondents were later excluded from the analysis because they were not willing to consume pasta featuring Opuntia, resulting in a final sample of 328 respondents. It is important to underline that the sample is not representative of the whole Italian population as found in other studies on consumer behaviors (e.g., [33,34,35,37]). 

### 2.2. Questionnaire

The survey was conducted using a structured questionnaire following the current literature about consumer behavior (e.g., [30,32]). It is important to underline that the study did not require ethics committee approval survey as in other consumer studies (e.g., [40]). The research followed the Italian National law (d.lgs. 196/2003) and following modifications by the EU. Prior to answer the questions, participants were briefly informed by research staff about the project that motivated the survey and their free decisions on their involvement with the research and assurance of no explicit or implicit coercion. Moreover, the information gathered for the present study is treated confidentially and the respondent identities are anonymous. All participants gave their informed consent before answering the questionnaire. The structured questionnaire was divided into four sections: (1) and (2) consider consumer habits about food and pasta choices, (3) considers consumer behaviors towards functional pasta featuring Opuntia, and (4) pertains to socioeconomic and demographic information. The first three sections asked questions with ten-point Likert scales with growing levels of evaluation (i.e., 1 denotes total disagreement and 10 denotes total agreement) as in other studies [32,34,35]. 

The first and the second sections of the questionnaire investigated respondent consumption characteristics, habits, and preferences for pasta in general (e.g., frequency of consumption, type of pasta consumed, places of consumption of pasta, preferences for pasta made with foreign durum wheat or for pasta made with Italian durum wheat, attributes of pasta worthy of attention etc.) [32]. Moreover, the attention people pay to the environmental- and health-related aspects of their food consumption behavior and also food neophobia and food technology neophobia have been considered, as highlighted by some authors [36,41].

The third part of the questionnaire focused on consumer behaviors towards functional pasta featuring Opuntia. According to similar studies concerning consumer behavior [34,35,41], participants were informed both according to the scientific literature [13,29] and the method used to make functional pasta featuring Opuntia, and that it is a good functional food for maintaining a normal body weight and the prevention of age-related metabolic disorders.

Respondent willingness to eat functional pasta containing Opuntia was evaluated by asking respondents to provide their positive or negative opinion about the question of “would you be willing to eat functional pasta containing Opuntia?” It is important to highlight that if participants were not willing to consume Opuntia then they would have been excluded from the analysis. Respondents were also asked to indicate their familiarity with eating functional pasta by questioning if they had ever heard about consuming functional pasta containing Opuntia before and their past consumption by asking if they had ever consumed functional pasta containing Opuntia in the past [35]. In addition, following [34], consumer perceptions for functional pasta containing Opuntia (i.e., disgust and environmental issues) and appreciation towards the nutritional content of functional pasta were investigated.

Finally, the fourth section contained detailed questions regarding the socioeconomic and demographic characteristics of respondents and their family (the questionnaire is available in the Supplementary Materials).

### 2.3. Methods

Factor analysis (FA) is commonly used to study consumers preferences and motivations with the aim to simplify the observed topic [32,42]. In other words, FA is a method used to study a topic by simplifying it into a smaller number of elements underlying a large number of detected variables [43]. In particular, FA allows the measurement of a latent variable which cannot be measured with a single variable, thus observing the relationship with a set of known variables [44].

In this study, a framework of preferences was drawn using FA with the answers of the questionnaire [32]. In other words, the FA technique with orthogonal rotation (varimax) was used to discover the main factors that could influence consumers to choose functional pasta containing Opuntia. Moreover, Keiser–Meyer–Olkin (KMO) and Barlett testing were applied in order to verify the sampling and correlation adequacy, while Kaiser’s criterion was applied to identify the appropriate number of factors to include in the analysis.

Hierarchical cluster analysis based on the FA results was later performed. The aim was to identify homogeneous groups with respect to a set of factors characterized by similarity and elements of difference amongst groups. Clusters were identified using Euclidean distances and Ward’s method [33,36]. 

All statistical elaborations were carried out using R (version 3.6.2, RStudio, Boston, MA, USA) [45].

## 3. Results

### 3.1. Sample Characteristics and Behaviors 

Participants for the study were conveniently sampled in the period of September–December in 2020. From an initial sample of 350 consumers, about 6% of respondents were excluded from the analysis as they were not willing to consume pasta containing Opuntia. This unwillingness (6% of the whole sample) was due to neophobia, technophobia, or disgust towards this new functional food. At the end, a total of 328 respondents were used for data analysis.

Table 1 shows some of the participants characteristics. In particular, the sample consists of more females (61.40% of the sample) than males, with a mean age of 50 years, a higher share of well-educated (88.60% of respondents) and married participants (64.47% of the sample). About 35% of the sample earned an income between 20,001–30,000 Euros per year, followed by 28% of respondents with an annual income between 30,001–40,000 Euros.

It is interesting to observe that 35% of the sample consumed pasta more than twice a week and about 59% of respondents consumed it at home (Table 2). Another important finding is that about 57% of the participants declared to alternate the consumption of durum wheat pasta with other types of pasta (e.g., stuffed pasta) once a month, while 55% of the sample claimed to consume fresh pasta and about 55% consumed egg pasta once a month. Moreover, some people claimed to never consume pasta with added vitamins (about 95% of the sample), cooked pasta (about 91%), frozen and then cooked pasta (about 90% of the respondents) and Kamut^®^ wheat pasta (Table 3). Finally, some people claimed to consume neither organic (64%) nor integral pasta (about 36% of the sample). It is important to underline that 100% of respondents had never consumed functional pasta containing Opuntia in the past. This is probably due to the unavailability of functional pasta on the market. Moreover, 95% of the sample had heard about eating functional pasta in the past.

With regard to the consciousness of Italians with respect to the origin of durum wheat, it is important to note that about 83.78% of the sample knew the origins of the raw materials. In particular, about 54% of respondents claimed that the pasta they consume is made with durum wheat cultivated in Italy (Table 4).

### 3.2. The Factors Explain Preferences of Respondents 

The KMO test result was equal to 0.81 and the Barlett’s test result (χ^2^ = 13.901; df = 3.741; *p*-value < 0.001) was significant [46], indicating that the sample and correlation matrix were appropriate for such analysis. Seven factors had eigenvalues over a Kaiser’s criterion of 1 and together explained 74% of the original variance. 

Table 5 shows the seven factors included in the analysis with their Cronbach’s α values. Moreover, following [32], we removed items with factor loading less than 0.45.

Factor analysis (FA) was used to identify the main preferences that could influence consumers in choosing functional pasta. It is important to consider that the sample was composed of people that were willing to consume pasta containing Opuntia. 

By reading Table 5, the seven main factors can be defined as observed below: 

Factor 1, “*health aspects*”, denotes a preference for the values of health and food tradition, as well as the nutritional and hygienic aspects of food, which are linked to both environmental and social issues and the food production methods. Another value underlying this factor is the one related to the seasonality of food that is directly linked to the environmental concerns [47].

Factor 2, “food neophobia” and factor 3, “food technology neophobia”, reflect people’s beliefs in their country’s culinary traditions. Similar results can also be seen for factor 4, “*types of pasta*”, which shows preferences for other types of pasta consumed than dry durum wheat (i.e., fresh pasta, stuffed pasta, and egg pasta) and reflects a tradition which is typical for local Italian products.

Factor 5, “*Italian pasta tradition*”, shows a preference for the basic Italian values of pasta in terms of the origin, territorial brand, and certification (i.e., quality, safety, and ethical certifications). Other values for this factor are related to both information about the health benefits of pasta, its production processes, and environmental impacts.

Factor 6, “*attributes of pasta*”, shows the attributes to which consumers pay attention when choosing pasta. This factor illustrates preferences for intrinsic and extrinsic characteristics of pasta. In this case, the preference shows a value of experience by consumers towards the product, that is the belief of cooking the typical Italian pasta.

Finally, factor 7, “*functional pasta*”, reveals potential preferences for functional pasta characteristics that are related with health and nutritional aspects, its potential price, its potential environmental impacts, and curiosity about this new food and the necessity to receive information about its production. Another value underlying this factor is the one related to customer loyalty to a pasta brand. In other words, the potential preference for pasta containing Opuntia might also be driven by customer loyalty to a brand and the price of the product.

### 3.3. Cluster Analysis: Potential Consumer Profiling

Cluster analysis allowed us to successfully and consistently identify seven clusters (R^2^ = 0.70) and the results are presented in Table 6. It is important to consider that the examined sample was composed of people that were willing to consume pasta containing Opuntia.

Cluster 1, “healthy and traditional consumers”, represented 7.71% of the sample. This group was characterized by well-educated people who paid attention to health characteristics and the social and environmental impacts of their food choices. Moreover, members of this group pay attention to traditions and the attributes of pasta but did not fear new food products. In fact, these consumers appeared to be neither neophobic nor technophobic, and their perceptions for functional pasta were positive in terms of both curiosity and attention towards the health, nutritional, and environmental aspects of this new food. 

Cluster 2, “refractory consumers”, represented about 6% of the sample. This group was characterized by educated people who were not neophobic or technophobic, and they did not pay attention to the health characteristics of their food choices, Italian traditions, or attributes of pasta. The perceptions of functional pasta were positive for this group.

“Neophobic and traditional consumers” formed cluster 3 and represented 4.75% of the sample. In this cluster, respondents were neophobic and they showed a low level of technophobia. They appeared sensitive to aspects such as the tradition, and intrinsic and extrinsic characteristics of pasta and they perceived functional pasta containing Opuntia in a negative light. Although consumers belonging to this cluster were neophobic, they did not perceive pasta containing Opuntia as a potential new food.

Cluster 4, “healthy, technophobic, and traditional consumers”, represented 43% of respondents. In this group, consumers were well-educated, technophobic, and paid great attention to the health and environmental impacts of their food choices. The perceptions towards of pasta containing Opuntia among consumers in this group appeared to be in line with the average level measured over the whole sample. They did not appear to be negative with respect to the themes proposed in the questionnaire (curiosity, healthy and nutritional aspects of functional pasta, its potential price, its potential environmental impacts, and the necessity to receive information about the production methods). 

Cluster 5, “healthy and expert consumers”, accounted for 28% of the sample. The cluster was associated with well-educated people who paid a large amount of attention to the health and environmental impacts of their food choices and attention to the intrinsic and extrinsic characteristics of pasta. This group featured the belief of cooking typical Italian pasta. Moreover, respondent perceptions for functional pasta were more positive than those of consumers belonging to the other groups.

“Neophobic and critical consumers“ formed cluster 6 and accounted for about 4% of the sample. In this cluster, respondents (with low education level) were neophobic but did not show a high level of technophobia and also paid attention to attributes of pasta; however, although the examined sample was composed of people that were willing to consume functional pasta, they appeared to negative consider the specific themes of the factor “functional pasta”. 

Similar perceptions were shown by consumers belonging to cluster 7, “neophobic consumers”, which represented 7% of respondents. Indeed, these consumers with a low education level appeared to be the most negative of all the groups with respect to the themes of “*functional pasta”*, although the whole sample was composed by people that were willing to consume functional pasta. Moreover, consumers belonging to cluster 7 had neophobic attitudes and did not pay attention to the health characteristic or social and environmental impacts of their food choices. 

## 4. Discussion

According to an Italian sample, this study has attempted to elucidate the main factors that could influence a consumer in choosing functional pasta containing Opuntia and to characterize distinct hypothetical consumer segments in terms of their food habits, pasta choices, and their perceptions towards functional pasta containing Opuntia. In particular, the preferences driving consumers to choose functional pasta containing Opuntia and hypothetical consumer segments have been identified here.

The study has been carried out with an Italian sample, since Italy is a reference country for the Mediterranean diet [34,48] and was the first country in the world to produce pasta [32], and it is also the second largest producer of Opuntia worldwide [23].

The study featured an explorative approach due to the non-representativeness of the sample to the Italian population. In fact, according to ISTAT (2020) [49], the average Italian is female, has a mean age of approximately 46 years (vs. 50 years for our sample), and a low education level (vs. a high education level for our sample). Nevertheless, the authors believe that this study offers the opportunity to add some new insights and to propose discussions regarding little-known food issues concerning the topic of consumer preferences and attitudes towards functional pasta containing Opuntia as a new food. 

The analysis showed very interesting results with respect to the understanding of both the perceptions of functional pasta containing Opuntia and also the main drivers of these perceptions.

The sample was composed of 328 respondents which were willing to consume pasta containing Opuntia, with 61.40% females, a mean age of 50 years, and a high education level (88.60% of respondents). The study confirmed the importance of pasta for Italian consumers and our results are consistent with the literature (e.g., [32]). In fact, according to Altamore et al. [32], pasta is a traditional food for the Italian population and people occasionally replace dried pasta with fresh pasta or other typical local pastas. In our case, in all identified clusters (except for cluster 5, “healthy and expert consumers”), people declared to alternate the consumption of durum wheat pasta once a month with other types of pasta, such as stuffed pasta, fresh pasta, and egg pasta (i.e., the “*type of pasta*”). 

According to some authors [50], consumers choose pasta on the basis of credentials (i.e., origin of raw material, brand, and price, etc.), while according to others authors [30] the origin, tradition, and healthy features of pasta lead consumers to have greater preferences for pastas that present ideal values for these attributes. In our case, the findings illustrate respondent preferences for the intrinsic and extrinsic features of pasta. In particular, the respondents choose pasta on the basis of characteristics like the origin of wheat, methods of production, nutritional characteristics, producer brand, type and color of pasta, time and type of cooking, quality certification, and price (i.e., “*attributes of pasta*”). In other words, the respondent preferences reflect the value of consumer experience towards the product, i.e., the belief to know and cook the typical Italian pasta. In fact, five out of seven clusters showed a good level of appreciation for pasta attributes (Table 6). The results highlight that consumers believe to be profoundly aware about which features a good pasta must have in order to be defined as a typical Italian pasta. In other words, consumer mindfulness governs the knowledge of this food under aspects such as external appearance, experience during preparation, and consumption etc. 

As mentioned above, the raw material origin is another important indicator for the quality of pasta for Italian consumers [32]. In our case, about 83.78% of the sample knew the origin of the wheat and, in particular, about 54% of respondents claimed that the pasta that they consume is made with durum wheat cultivated in Italy. Similar results were found reached by Altamore et al. [32], who found that Italian consumers preferred to eat pasta with only Italian grain or alternatively with grains cultivated entirely in Southern Italy. Moreover, the findings showed preferences for the basic Italian characteristics of pasta in terms of the origin, territorial brand and quality, safety, and ethical certifications (i.e., the ”*Italian pasta tradition*”). According to some authors [51], labels are frequently known as useful tools to help customers in the food selection decision-making process. Besides, information provided on the label gives consumers the opportunity to make choices more consciously and make them aware regarding the complex aspects of consumption [52] and the environmental, social, and ethical aspects of the product. In our case, people belonging to three out of seven clusters (i.e., cluster 1, “healthy and traditional consumers”, cluster 3, “neophobic and traditional consumers”, and cluster 4, “healthy, technophobic, and traditional consumers”) claimed a need during purchase to receive information about the pasta production processes, origin of durum wheat, information about the quality, safety, and ethical issues, and the product’s environmental impact (i.e., the ”*Italian pasta tradition*”).

According to Defrancesco et al. [31] people are looking not only for nutritional features of pasta but also for new characteristics like health and environmental features that express a perceived quality. Also, in our case, the sample showed an underlying preference for the values of healthy and tradition of food, as well as nutritional and hygienic aspects which were linked to both environmental and social issues and production methods (i.e., “*health, aspects*”). According to Altamore et al. [32], Italian people who values healthiness also value sustainability, and these values in consumer perceptions are linked to the origin of durum wheat and in particular to the Italian regions where durum wheat is traditionally cultivated. Moreover, our results are consistent with other authors [53,54] which have shown that the interest levels for educated individuals in functional foods are higher than others. In particular, highly educated consumers are prone to look for foods which provide greater health benefits [53,54]. In our case, five out of seven clusters were composed of well-educated respondents and among them three clusters were composed of respondents who paid attention to elements such as the health and tradition characteristics, as well as nutritional and hygienic. In particular, in cluster 5, “healthy and expert consumers”, well-educated respondents paid attention to healthy aspects of food and to the intrinsic and extrinsic characteristics of pasta. This cluster appeared to be the most positive of all the groups with respect to the themes proposed in the factor *“health aspects”*.

It is important to observe that even if our results reflect people’s membership in their country’s culinary traditions due to the neophobic and technophobic aspects highlighted in the sample (factors 2 and 3), our findings (four out of seven clusters, i.e., clusters 1, 2, 4, and 5) are consistent with the current literature [55], in which it has been shown that low food neophobia, together with familiarity with a new ingredient, increase consumer acceptance. It is important to consider that 95% of the sample had heard about the consumption of functional pasta containing Opuntia in the past and thus they showed familiarity with this new type of pasta. Moreover, among the clusters composed of respondents with a high education level, two consumers groups (i.e., clusters 3 and 4) were composed of neophobic and technophobic people, respectively; however, they appeared to be positive in terms of the consideration of aspects of functional food containing Opuntia as proposed in the questionnaire (curiosity, health, and nutritional aspects of functional pasta, along with its potential price, potential environmental impacts, and the necessity to receive information about its production). In particular, in the case of cluster 3, “neophobic and traditional consumers”, although consumers were neophobic and did not pay attention to healthy aspects of food, they did not perceive pasta containing Opuntia as a new food. These findings could be due to Opuntia being used as an ingredient in [56] some Southern Italian recipes (such as Sicily, Sardinia, and Campania) and in some Italian food events [57]. Thus, this familiarity with a new ingredient increases consumer acceptance [55], as highlighted in other studies about consumer behaviors (see e.g., [36]). In cluster 4, although consumers were technophobic they did not perceive pasta containing Opuntia as a new food. These results could be due to respondents propensity to pay attention to healthy aspects of food (one of the themes proposed both in the factor titled *“health aspects”* and the factor titled *“functional pasta”*). This may also pertain to their familiarity with to the use of Opuntia in some Italian recipes [56] and some Italian food events [57]. On the other hand, the highest food neophobia scores showed in the clusters 6 and 7 (“neophobic and critical consumers” and “neophobic consumers”, respectively) were due to the low education of participants, as seen in other studies regarding consumer behavior (e.g., [41]). Moreover, although the whole sample was composed of people that were willing to consume pasta containing Opuntia, respondents belong to cluster 6 and 7 appeared to be negative with respect to specific themes of the factor “functional pasta” (i.e., the healthy and nutritional aspects of functional pasta and the necessity to receive information about its production method), while they were positive towards other functional pasta aspects (i.e., the potential price). These findings were due to low annual income (<10,000 Euros per year and 10,001–20,000 Euros per year). It is important to observe that cluster 6 and 7 were differentiated in terms of respondent attention to pasta attributes. In fact, consumers belonging to cluster 6 were more careful regarding pasta attributes.

Moreover, the proposal of unusual food (as functional pasta containing Opuntia) emphasizes the need to understand consumer preferences and their expectations as a starting point for production [58]. In this context, people’s expectations are important factors of new product acceptance [59]. The findings showed a lot of interest among respondents in terms of health benefits, and the nutritional and environmental aspects of functional pasta should encourage acceptance and the consumption of this new functional food (i.e., factors relating to “*functional pasta*”). In fact, five out of seven clusters (i.e., clusters 1, 2, 3, 4, and 5) were composed of well-educated respondents who were interested in the functional pasta aspects and, among them, three clusters (i.e., clusters 1, 4, and 5) were composed of people who paid attention to elements such health aspects and tradition, along with the nutritional and hygienic aspects, social and environmental impacts, and production methods. These results have been confirmed by other studies regarding new food (see e.g., [33,36]), which have reported that a high education level and information regarding the organoleptic and nutritional characteristics of a new food are important aspects for any attempt to increase market acceptance. Moreover, the findings have highlighted, in agreement with other studies about Italian consumer preferences towards new food [33,34,35,36,39], that information about the production method and curiosity towards this new food could drive product acceptance. According to Altamore et al. [32], one of the aspects which Italian consumers rely on to evaluate pasta is the place and method of production, as well as the product price. In our case, respondent preferences for pasta containing Opuntia could be driven by its price (i.e., “*functional pasta*”). In fact, respondents belonging to clusters 6 and 7 appeared to have a positive behavior towards some functional pasta aspects because of the potential price. 

## 5. Conclusions

Consumer attitudes and perceptions towards new foods are important to consider when attempting to introduce said foods. Besides, the involvement of consumers in the process of new product development is an important factor to consider for the design of new foods. Moreover, new products also represent potential revenue for food companies. 

Although the sample used in this research cannot be considered to be representative of the entire Italian population due to the explorative approach of the study, like in many research studies about consumer behavior, the obtained results provide interesting hints to understand the process of consumer decision-making in this regard. In fact, further studies are necessary to better understand the acceptance of Italian consumers towards functional pasta containing Opuntia in terms of their individual preferences, attitudes, or concerns.

This study has attempted to provide insights and discuss Italian consumer preferences for functional pasta containing Opuntia using locally available cactus as an ingredient that is individually and culturally accepted. Seven profiles of Italian consumers were identified in order to develop a better understanding of consumers’ opinions and to facilitate the design of marketing strategies. The main findings show that the role of perception among people depends on their education level. In fact, although our clusters were characterized for different variables, some interesting aspects arose, namely, clusters associated with respondents with a high education level differed from those with a low education level. Among clusters composed of respondents with a high education level, two consumers groups were composed of neophobic and technophobic people, respectively; however, they appeared to be positive toward aspects of functional food containing Opuntia. Moreover, the findings show that the health benefits and nutritional and environmental aspects of functional pasta should encourage the acceptance and consumption of this new functional food. In addition, the respondent preferences reflect a value of experience towards pasta, i.e., the belief of cooking the typical Italian pasta. This means that Opuntia use for functional pasta production should maintain the organoleptic and physical properties of durum wheat-based pasta. In addition, respondent preferences for pasta containing Opuntia could also be driven by the product price.

Therefore, from the results obtained in this study, it is possible to develop marketing strategies considering both positive consumer perceptions regarding the use of *Opuntia ficus-indica* in pasta production and the criteria used by Italian consumers to evaluate pasta; however, it is important to keep the explorative approach of the study in mind. 

## Figures and Tables

**Table 1:** Socioeconomic and demographic characteristics of the sample (*n* = 328).

Variables	%
Gender	
Male	38.60
Female	61.40
Total	100.00
Education	
Low education	11.40
High education	88.60
Total	100.00
Status	
Unmarried	23.68
Married	64.47
Separated/divorced	11.85
Total	100.00
Annual income (Euros)	
<10,000	4.39
10,001–20,000	13.60
20,001–30,000	35.08
30,001–40,000	28.07
40,001–50,000	11.84
>50,001	7.02
Total	100.00

Source: Our elaboration on survey data.

**Table 2:** Frequency of durum wheat pasta consumption and places of consumption.

Variables	%
Frequency of consumption	
never	2.19
Once a month	5.26
Once a week	14.91
Twice a week	20.18
More than twice a week	35.08
Every day	22.38
Total	100.00
Places of consumption	
At home	58.77
Out of home (bars and restaurants)	2.19
Both	39.04
Total	100.00

Source: Our elaboration on survey data.

**Table 3:** Periodicity of the consumption of other pasta types.

Frequency of Consumption	Integral Pasta	Fresh Pasta	Organic Pasta	Stuffed Pasta	Egg Pasta	Frozen Pasta Cooked	Pasta Cooked	Pasta with Added Vitamins	Kamut^®^ Wheat Pasta
Never	35.96	13.60	64.03	25.88	18.42	89.91	90.79	94.74	81.58
Once a month	32.89	55.26	21.93	57.46	54.82	8.33	7.02	2.63	14.03
Once a week	17.54	28.95	7.01	15.35	25.00	0.88	1.32	2.19	3.07
Twice a week	6.14	1.75	2.19	0.88	1.32	0.44	0.87	0.44	0.88
More than twice a week	3.95	0.44	3.51	0.43	0.44	0.00	0.00	0.00	0.44
Every day	3.52	0.00	1.33	0.00	0.00	0.44	0.00	0.00	0.00
Total	100.00	100.00	100.00	100.00	100.00	10.00	100.00	100.00	100.00

Source: Our elaboration on survey data.

**Table 4:** Knowledge of the origin of durum wheat used to make the consumed pasta.

Variables	%
I do not know	16.22
Wheat cultivated only in Italy	53.96
Wheat cultivated only in Southern Italy	8.33
Wheat cultivated abroad	0.00
Wheat cultivated both in Italy and abroad	21.49
Total	100.00

Source: Our elaboration on survey data.

**Table 5:** Factor analysis with a varimax rotation.

	Factor 1	Factor 2	Factor 3	Factor 4	Factor 5	Factor 6	Factor 7
*Health aspects* (α = 0.95) **							
Health effects	0.87						
Proteic	0.78						
Caloric	0.86						
Fat	0.84						
Hygienic	0.84						
Geographic	0.76						
Seasons	0.77						
Traditions	0.69						
Environmental impact	0.80						
Social impact	0.77						
Production method	0.76						
*Food neophobia* (α = 0.99) **							
New *		0.72					
Different culture *		0.95					
New food *		0.78					
Ethnic restaurant *		0.82					
*Food technology neophobia* (α = 0.76) **							
No technology			0.74				
Environmental benefits overestimated			0.88				
World hunger overestimated			0.87				
Low quality			0.77				
Good enough			0.75				
Unknown effect on health			0.56				
*Type of pasta* (α = 0.75) **							
Fresh pasta				0.57			
Stuffed pasta				0.66			
Egg pasta				0.62			
*Italian pasta tradition* (α = 0.91) **							
Origin as quality indicator					0.64		
Italian wheat					0.69		
Southern Italy wheat					0.52		
Recycled packaging					0.60		
Info health benefits					0.75		
Info production					0.82		
Territorial brand					0.80		
Low impact					0.86		
Quality certification					0.85		
Health certification					0.83		
Ethical certification					0.81		
*Attributes of pasta* (α = 0.91) **							
Method						0.73	
Type						0.73	
Origin						0.80	
Pasta brand						0.73	
Labeled nutrition						0.82	
Color						0.68	
Time						0.67	
Cooking method						0.63	
Quality certification						0.77	
Price						0.48	
*Functional pasta* (α = 0.87) **							
Curiosity							0.74
Health benefits							0.86
More nutrients							0.81
Low environmental impact							0.89
Low cost							0.58
Pasta factory							0.62
More info							0.83

* Reversed coded. ** The questionnaire is available in the Supplementary Materials, along with details about the abbreviations of the variables.

**Table 6:** Profiles of consumers segments (*n* = 328) with mean scores of variables within the groups.

*n*	Label of Consumers Profiles	%	Edu	Food Consumption Attitudes			Pasta Consumption Attitudes			
				Health Aspects	Food Neophobia	Food Technology Neophobia	Type of Pasta	Italian Pasta Tradition	Attributes of Pasta	Functional Pasta
1	Healthy and traditional consumer	7.71	0.74	7.36	4.55	4.09	1.10	7.36	6.83	5.80
2	Refractory consumer	5.56	0.58	5.29	3.15	2.20	1.07	3.89	4.87	5.55
3	Neophobic and traditional consumer	4.75	0.51	2.29	6.86	3.18	1.35	7.14	7.34	6.31
4	Healthy, technophobic, and traditional consumer	43.23	0.60	8.39	4.59	6.51	1.10	8.70	8.34	7.33
5	Healthy and expert consumer	28.10	0.94	8.73	5.62	6.03	0.44	3.16	9.38	8.02
6	Neophobic and critical consumer	3.64	0.11	4,23	8.28	5.43	0.71	3.87	5.67	3.14
7	Neophobic consumer	7.01	0.28	2.84	8.73	3.08	1.10	2.71	2.34	2.29
	The whole sample	100.00	0.90	6.58	5.17	4.41	1.07	6.67	6.68	6.77

Legend: Edu = education of respondent: 0 = low education; 1 = high education level. For health aspects, food neophobia, food technology neophobia, Italian pasta tradition, attributes of pasta, and functional pasta, ten-point Likert scales have been used (1: totally disagree, to 10: totally agree). The periodicity values for other types of pasta are coded as 0 = never; 1 = once a month; 2 = once a week; 3 = twice a week; 4 = more than twice a week; 5 = every day.

## Supplementary Materials

The following are available online at https://www.mdpi.com/article/10.3390/foods10040803/s1, Table S1: Variables used in the questionnaire.
