# Supplementary material for: Opuntia ficus-indica as an Ingredient in New Functional Pasta: Consumer Preferences in Italy
Source: Foods. 2021 Apr 8;10(4):803. doi: 10.3390/foods10040803 (PMC8068382; doi:10.3390/foods10040803)
Supplement: Supplementary file 1 [file foods-10-00803-s001.pdf]

The questionnaire

Table S1. Variables used in the questionnaire.

Item groups	Items	Issues	*Source*
		**Section 1: consumers' habits about food (mean score on 10-point scale)**	
1		Responsibility for the grocery shopping in family (0=no, 1=yes)	
2	Aspects you pay attention when you consume food (*Health aspects*)	The hygienic requirements of food (hygienic)	[1–8]
		The effects on human health (health_effects)	
		The caloric intake of food (caloric)	
		The food protein intake (proteic)	
		The food fat intake (fat)	
		The environmental impact of food consumption (environmental_impact)	
		The geographic origin of food (geographic)	
		The food and culinary traditions (traditions)	
		The social impact of food consumption (social_impact)	
		The production method of food (production_methodt)	
		The seasonality of food (seasons)	
3	Agreement or disagreement with the following statements (*Food neophobia*)	I am very particular about the foods I will eat (attention)	[9]
		I like to try new ethnic restaurants (ethnic_restaurant*)	
		If I don't know what is in a food, I won't try it" (no_try)	
		I am constantly sampling new and different foods (new*)	
		I like foods from different cultures (different_culture*)	
		At dinner parties, I will try a new food (new_food*)	
		Ethnic food looks too weird to eat (strange)	
		I don't trust new food (do not trust)	
		I am afraid to eat things I have never had before (fear)	
4	Agreement or disagreement with the following statements (*Food technology neophobia*)	I don't have enough knowledge on effects of new food technologies on human health (don't_know_effect on health)	[9]
		New food technologies decrease the natural quality of food (low_quality)	

		There is no sense in trying out high-tech food products because the ones I eat are already good enough" (good_enough)	
		The benefits of new food technologies to reduce world hunger are often overstated (world_hunger_overstimated)	
		The environmental benefits of new food technologies are often overstated (environmental_benefits_overstimated)	
		New food technologies are unnecessary (no_tecnology)	
		**Section 2: consumers' habits about pasta choices**	
5	Frequency of durum wheat pasta consumption (0= never; 1=once a month; 2=once a week; 3= twice a week; 4= more than twice a week; 5= every day)	never	[1]
		once a month	
		once a week	
		twice a week	
		more than twice a week	
		every day	
6	Periodicity of other pasta types consumption (0= never; 1=once a month; 2=once a week; 3= twice a week; 4= more than twice a week; 5= every day) (*Type of pasta*)	organic pasta	[1]
		integral pasta	
		fresh pasta	
		stuffed pasta	
		egg pasta	
		frozen pasta cooked	
		pasta cooked	
		pasta with added vitamins	
		Kamut® wheat pasta	
7	Places of consumption	at home	[1]
		out of home -bars, restaurants	
		both	
8	Knowledge of origin of durum wheat used to make the consumed pasta	I don't know	[1]
		wheat cultivated only in Italy	
		wheat cultivated only in the Southern Italy regions	
		wheat cultivated abroad	
		wheat cultivated both in Italy and abroad	
9	Agreement or disagreement with the following statements (*Italian pasta tradition*)	The origin of the wheat is a qualitative indicator for Pasta (origin as quality indicator)	[1]
		I preference to consume pasta produced with durum wheat cultivated only in Italy (Italian_wheat)	
		I preference to consume pasta produced with durum wheat cultivated only in the South of Italy (Southern_Italy_wheat)	
		I believe Italian pasta is produced only with Italian durum wheat (Italy)	
		I believe Italian pasta is produced with durum wheat cultivated both in Italy and abroad (Italy_and_abroad)	
		I preference to buy pasta with recyclable packaging (recycle_packaging)	
		I believe it is important to have information about benefits effect of pasta (info_health_benefits)	
		I believe it is important to have information about production process of pasta (info_production)	
		I prefer to buy Italian pasta brands regardless of the origin of durum wheat (no_origin)	
		I prefer to buy pasta with a brand linked to an Italian territory with traditions of durum wheat cultivation (territorial_brand)	
		I prefer to buy pasta low environmental impact (low_impact)	
		I prefer to buy pasta with quality certifications (i.e. organic certification etc) (quality_certification)	
		I prefer to buy pasta with healthy certifications (healthy_certification)	
		I prefer to buy pasta with ethical certifications (ethical_certification)	
10	Attributes you pay attention when you choose pasta (*Attributes of pasta)*	Production method (method)	[1]
		Pasta types (type)	
		Origin of durum wheat (origin)	
		Producer brand (pasta_brand)	
		Nutritional information on label (label_nutrition)	
		Color (color)	
		Cooking time (time)	
		Cooking type (cook_type)	
		Quality certifications (quality_certification)	
		Price (price)	
11		**Section 3: consumers' behavior towards functional pasta comprising Opuntia (mean score on 10-point scale)**	
12		Have you ever heard about functional pasta comprising Opuntia? (Fam) (0=No; 1=Yes)	[9,10]
13		Have you ever eaten functional pasta comprising Opuntia before? (Cons) (0=No; 1=Yes)	
14		Would you be willing to eat functional pasta comprising Opuntia? (Willing) (0=No; 1=Yes)	
15	What functional pasta characteristics could affect your decision to eat it? (*Functional pasta*)	If I think to eat functional pasta comprising Opuntia, I'm curious to try it (curiosity)	[1,9]

		If functional pasta comprising Opuntia produces health benefits, I would eat it (health_benefits)
		If functional pasta comprising Opuntia are more nutrient than conventional pasta, I would eat it (more_nutrient)
		If functional pasta production is less impactful than conventional pasta, I would eat it (low_env_impact)
		If functional pasta is less expensive than conventional pasta, I would eat it (low_cost)
		If functional pasta is produced by my trusted pasta manufacturing, I would eat it (pasta_factory)
		If I get more information about functional pasta comprising Opuntia, I would eat it (more_info)
		I'm disgusted to the idea of eating functional pasta comprising Opuntia (disgust*)
	**Section 4: sociodemographic information**	
16		Age
17		Gender (0=female; 1=male)
18		Education : 0=primary or secondary (low education); 1=degree, master and/or PhD (high education)
19		Status (0= unmarried; 1= married; 2= separated/divorced)
20		Annual Income (0=< 10,000; 1=10,001 – 20,000; 2=20,001 – 30,000; 3=30,001 – 40,000; 4=40,001 – 50,000; 5=> 50,001)

*Reversed coded.

Word in brackets refers to the abbreviation of the variable in the manuscript.

References

1. Altamore, L.; Ingrassia, M.; Columba, P.; Chironi, S.; Bacarella, S. Italian Consumers' Preferences for Pasta and Consumption Trends: Tradition or Innovation? *J. Int. Food Agribus. Mark.* **2019**, *0*, 1–24.
2. Palmieri, N.; Perito, M.A.; Lupi, C. Consumer acceptance of cultured meat: some hints from Italy. *Br. Food J.* **2020**.
3. Palmieri, N.; Perito, M.A.; Macrì, M.C.; Lupi, C. Exploring consumers' willingness to eat insects in Italy. *Br. Food J.* **2019**, *121*.
4. Palmieri, N.; Forleo, M.B. The potential of edible seaweed within the western diet. A segmentation of Italian consumers. *Int. J. Gastron. Food Sci.* **2020**, 100202.
5. Palmieri, N.; Simeone, M.; Russo, C.; Angela, M. Profiling young consumers ' perceptions of GMO products : A case study on Italian undergraduate students. *Int. J. Gastron. Food Sci.* **2020**, *21*, 100224.
6. Palmieri, N.; Suardi, A.; Pari, L. Italian consumers' willingness to pay for eucalyptus firewood. *Sustain.* **2020**, *12*.
7. Palmieri, N.; Suardi, A.; Latterini, F.; Pari, L. The eucalyptus firewood: Understanding consumers' behaviour and motivations. *Agric.* **2020**, *10*, 1–12.
8. Palmieri, N.; Perito, M.A. Consumers' Willingness To Consume Sustainable and Local Wine in Italy. *Ital. J. Food Sci* **2020**, *32*, 222–233.
9. Verbeke, W. Profiling consumers who are ready to adopt insects as a meat substitute in a Western society. *Food Qual. Prefer.* **2015**, *39*, 147–155.
10. Bouazizi, S.; Montevecchi, G.; Antonelli, A.; Hamdi, M. Effects of prickly pear (Opuntia ficus-indica L.) peel flour as an innovative ingredient in biscuits formulation. *LWT* **2020**, *124*.
